# Doxorubicin-Induced Cardiotoxicity: A Comprehensive Update

**DOI:** 10.3390/jcdd12060207

**Published:** 2025-05-30

**Authors:** Vasvi Bhutani, Fahimeh Varzideh, Scott Wilson, Urna Kansakar, Stanislovas S. Jankauskas, Gaetano Santulli

**Affiliations:** 1 Department of Medicine (Division of Cardiology), Wilf Family Cardiovascular Research Institute, Einstein Institute for Aging Research, Albert Einstein College of Medicine, New York City, NY 10461, USA; vasvi.buthani@einsteinmed.edu (V.B.); fahimeh.varzideh@einsteinmed.edu (F.V.); scott.wilson@einsteinmed.edu (S.W.); urna.kansakar@einsteinmed.edu (U.K.); stanislovas.janksuskas@einsteinmed.edu (S.S.J.); 2 International Translational Research and Medical Education (*ITME*) Consortium, Academic Research Unit, Department of Advanced Biomedical Sciences, “Federico II” University, 80131 Naples, Italy; 3 Department of Molecular Pharmacology, Einstein-Mount Sinai Diabetes Research Center (*ES-DRC*), Einstein Institute for Neuroimmunology and Inflammation (*INI*), Fleischer Institute for Diabetes and Metabolism (*FIDAM*), Albert Einstein College of Medicine, New York City, NY 10461, USA

**Keywords:** anthracyclines, cardiomyopathy, cell death, DNA damage, doxorubicin, heart failure, oncology

## Abstract

Doxorubicin is an anthracycline chemotherapeutic that is widely used for treating various malignancies, including breast cancer, lymphomas, and sarcomas. Despite its efficacy, its clinical utility is limited by a well-documented risk of cardiotoxicity, which may manifest acutely or chronically. Doxorubicin works by intercalating DNA and inhibiting topoisomerase II, leading to DNA damage and cell death. However, this mechanism is not selective to cancer cells and can adversely affect cardiac myocytes. The introduction of doxorubicin into oncologic practice has revolutionized cancer treatment, but its cardiotoxic effects remain a significant concern. This systematic review aims to comprehensively examine the multifaceted impact of doxorubicin on cardiac structure and function through both preclinical and clinical lenses.

## 1. Introduction

Doxorubicin is an effective anticancer agent that is widely used to treat breast cancer and other malignancies [1,2,3,4,5,6,7,8,9]. Unfortunately, a common side effect of doxorubicin is cardiotoxicity, which can cause heart failure and, ultimately, death [2,9,10,11]. The therapy carries a 3- to 5-fold greater risk for cardiotoxicity than other cancer drugs [1,12,13,14,15]. According to the most recent statistics, up to 48% of patients receiving doxorubicin can develop heart failure [3,4,16].

## 2. Doxorubicin: Pharmacokinetics and Pharmacodynamics

Doxorubicin is a well-established anthracycline antibiotic, exerting its anticancer effects primarily through two key mechanisms: DNA intercalation and topoisomerase II inhibition. The drug intercalates between DNA base pairs, disrupting the DNA double helix structure, which consequently hampers DNA replication and transcription processes [17]. This intercalation induces significant alterations in the chromatin configuration, enhancing the susceptibility of DNA to cleavage [18]. Moreover, doxorubicin acts as a poison to topoisomerase II, an enzyme crucial for DNA unwinding, thereby preventing the relaxation of supercoils that occur prior to DNA replication [19,20]. This inhibition results in double-strand breaks in the DNA, particularly at regions of active transcription, ultimately leading to cellular death. Additionally, doxorubicin is known to promote oxidative stress through the generation of free radicals, further contributing to cellular damage. This oxidative environment can damage cellular components, including proteins and lipids, affecting overall cellular viability [21]. The cumulative effect of these mechanisms leads to pronounced cytotoxicity in rapidly dividing cancer cells while unfortunately also being implicated in cardiotoxicity [22,23]. Thus, the dual action of intercalation and enzymatic inhibition, alongside its propensity to induce oxidative stress [24,25], constitutes a multifaceted approach that accounts for the efficacy of doxorubicin as a chemotherapeutic agent.

The pharmacokinetic of doxorubicin is complex and characterized by its rapid distribution, metabolism, and elimination, typically exhibiting three-compartment model behavior. After intravenous administration, doxorubicin reaches peak plasma concentrations within minutes, followed by a swift distribution phase [26]. The drug is extensively bound to plasma proteins, primarily albumin and α1-acid glycoprotein, with less than 5% remaining unbound in circulation [27]. This high protein binding affects its distribution and bioavailability, as well as its elimination half-life, which can vary significantly among individuals due to factors such as age and genetic polymorphisms [28]. The volume of distribution of doxorubicin is extensive, indicating significant tissue uptake and particular accumulation in organs like the liver, spleen, kidneys, and lungs [26]. Following metabolic activation in the liver, doxorubicin is primarily metabolized to its active metabolite, doxorubicinol, which also contributes to the cytotoxic effects of the drug, albeit with associated cardiotoxic risks [26]. The elimination phase of doxorubicin involves renal clearance, but a substantial fraction is excreted in bile as metabolites, underpinning its complex excretion pathway [29,30]. Due to variations in pharmacokinetic parameters such as clearance and half-life across diverse patient populations and to formulation differences of the drug (e.g., liposomal vs. conventional), an understanding of the pharmacokinetics of doxorubicin is crucial for optimizing its therapeutic use while minimizing toxicity [30,31,32].

## 3. Doxorubicin in Cardiac Tissue

With its poor excretion and strong affinity for cardiac tissue, doxorubicin and its metabolites accumulate within the myocardium. Upon administration, doxorubicin readily penetrates myocardial cells, where it interacts with cardiolipin, a phospholipid predominant in the inner mitochondrial membrane. This interaction facilitates passive diffusion of doxorubicin into cardiomyocytes [33]. The consequence of its accumulation is a toxic environment within the heart, significantly impacting mitochondrial function, respiration, and the balance of reactive oxygen species (ROS) [34]. Doxorubicin metabolites, including doxorubicinol, exacerbate an already precarious situation by enhancing oxidative stress and mitochondrial dysfunction, which are pivotal in the pathogenesis of drug-induced cardiomyopathy [35]. These mechanisms lead to an increase in lipid peroxidation levels, reduced mitochondrial membrane potential, and subsequent cardiomyocyte apoptosis, effectively compromising cardiac health [31,35,36]. Recent studies indicate that the alterations triggered by doxorubicin are not merely acute; chronic exposure can result in progressive damage that manifests as cardiac remodeling, hypertrophy, and, ultimately, congestive heart failure [37,38]. Understanding the metabolic pathways through which doxorubicin exerts its effects in cardiac tissue is crucial for recognizing both its acute cardiotoxicity and the delayed effects observed after treatment. Various protective strategies have been explored, such as antioxidant therapies and agents that modulate mitochondrial function, to mitigate these toxicities [39]. Additionally, pharmacological interventions targeting specific pathways, such as the PI3K pathway, have shown promise in preserving cardiomyocyte integrity and function during doxorubicin therapy [33,40]. For instance, Astragalus polyphenols and curcumin are compounds with such cardioprotective potential via acting on the PI3K pathway indicated in cellular survival and defense against oxidative stress [40,41]. Continued investigation into the cardiotoxic mechanisms of doxorubicin, particularly regarding its impacts on mitochondrial dynamics and signaling pathways, and into concurrent therapies to minimize these effects remains vital for enhancing patient safety during cancer treatment.

## 4. Acute and Chronic Cardiotoxicity

Doxorubicin-induced cardiotoxicity is classified temporally into acute (occurring within a week of administration) and chronic (months to years post-treatment). Acute toxicity includes transient ECG abnormalities, arrhythmias, and pericarditis. Chronic toxicity manifests as progressive left-ventricular dysfunction, often culminating in symptomatic heart failure. Cumulative dose is the strongest predictor of cardiotoxicity, with risk increasing sharply beyond 450–550 mg/m^2^. Chronic cardiotoxicity results from persistent injury to cardiac myocytes, including DNA damage, impaired mitochondrial function, and activation of apoptotic pathways. It may be possible to detect such subclinical myocardial dysfunction by advanced imaging or biomarker changes before overt clinical symptoms develop.

Acute cardiotoxicity has an incidence of ~11% and typically manifests within days after starting doxorubicin treatment, whilst chronic cardiotoxicity usually ensues weeks or months after having completed the doxorubicin treatment [42,43,44,45,46,47]. Hence, cardiac injury can occur as early as a single dose of doxorubicin or become evident as symptomatic heart failure months after doxorubicin treatment. However, since doxorubicin cardiotoxicity is dose-dependent, effective breast cancer treatment is frequently hampered by using doxorubicin at lower than therapeutically suitable doses.

## 5. Doxorubicin and Cardiomyocyte Death

Numerous mechanisms have been reported to contribute to doxorubicin-induced cardiomyocyte death, including oxidative stress, mitochondrial dysfunction, dysregulation of calcium homeostasis, activation of apoptotic pathways, and engagement of the endoplasmic reticulum (ER) stress response. The extent of myocyte loss correlates with cardiac functional impairment and long-term prognosis.

A direct toxicity of doxorubicin on cardiomyocytes, eventually leading to their necrosis, is considered one of the most relevant mechanisms of heart failure in doxorubicin-treated patients [48,49,50]. In fact, substantial evidence indicates that necrosis is significantly augmented in doxorubicin-treated hearts and is mainly the result of increased oxidative stress and cellular damage leading to cell swelling, membrane rupture, and, ultimately, cell lysis [44,51,52,53,54,55,56,57].

Doxorubicin induces necrosis predominantly through the generation of ROS and calpain-mediated proteolysis of structural proteins in cardiomyocytes. Elevated intracellular ROS levels result from the interaction of doxorubicin with cellular components, leading to oxidative damage and mitochondrial dysfunction [58,59,60,61]. Mitochondrial injury is a hallmark of necrosis, where doxorubicin promotes the opening of the mitochondrial permeability transition pore (MPTP), resulting in depolarization, loss of ATP production, and eventual cell death [62]. Upon treatment with doxorubicin, cardiomyocytes exhibit significant swelling and rupture indicative of necrosis, often correlating with increased release of lactate dehydrogenase (LDH), a marker for necrotic cell death, into the extracellular space [63].

Apoptosis is another pathway through which doxorubicin promotes cardiomyocyte death [64]. The activation of various apoptosis signaling pathways occurs via the accumulation of ROS and activation of pro-apoptotic factors [65]. Doxorubicin induces both intrinsic and extrinsic apoptotic pathways. The intrinsic pathway is activated by the release of cytochrome c and other apoptogenic factors from damaged mitochondria [66], while the extrinsic pathway is promoted by upregulation of death receptors such as Fas [49,67,68]. The activation of these pathways is often exacerbated by the transcriptional changes induced by doxorubicin, which upregulate pro-apoptotic factors and downregulate anti-apoptotic proteins, leading to an increased propensity for cardiomyocyte death [69]. Specifically, the depletion of GATA4, a critical survival factor for cardiomyocytes, significantly enhances apoptosis [23]. The final execution of apoptosis is typically characterized by the activation of caspases, particularly caspase-9 and -3, mediating cellular condensation and fragmentation into apoptotic bodies [70,71].

Emerging research highlights other non-classical forms of cell death that may come into play in doxorubicin-induced cardiomyocyte toxicity. For instance, necroptosis, a regulated form of necrosis, has been suggested to contribute to cardiomyocyte death, potentially triggered by the engagement of receptor-interacting protein kinases (RIPK1 and RIPK3) following doxorubicin exposure [49]. Furthermore, ferroptosis, characterized by iron-dependent lipid peroxidation, represents another potential mechanism that might mediate cardiomyocyte death in the context of doxorubicin treatment [72,73,74,75,76,77,78,79,80,81,82,83,84,85,86,87,88], as the drug exacerbates oxidative stress within cells [74,89,90,91,92,93,94].

Endoplasmic reticulum stress, characterized by the accumulation of misfolded proteins, plays a crucial role in doxorubicin-induced toxicity. This stress response activates pathways that lead to apoptotic cell death if homeostasis cannot be restored [95,96]. Increased expression of ER stress markers, such as GRP78, is observed in response to doxorubicin administration, suggesting a shift in protein homeostasis that culminates in cell death [97,98,99].

Recent insights have identified autophagic dysregulation as an additional factor in doxorubicin-induced cardiotoxicity [82,99,100,101,102,103,104,105,106,107,108,109,110,111,112]. Impaired autophagic flux is associated with increased cardiomyocyte apoptosis following doxorubicin treatment, indicating a failure in the cellular quality control mechanisms necessary for maintaining myocardial health [113]. Pharmacological interventions targeting the pathways of mitochondrial dysfunction, oxidative stress, and apoptosis could provide effective strategies to mitigate the cardiac damage associated with doxorubicin therapy [114,115,116].

## 6. Oxidative Stress

Doxorubicin undergoes redox cycling in the body in the presence of iron, producing superoxide anions, hydrogen peroxide, and hydroxyl radicals. These reactive species damage cellular components, including lipids, proteins, and nucleic acids, which is especially detrimental to mitochondrial function. Cardiac cells are particularly vulnerable to such oxidative insults due to their inherently low levels of endogenous antioxidants, such as catalase and superoxide dismutase, which are crucial for mitigating oxidative stress. The presence of iron–doxorubicin complexes exacerbates oxidative injury by catalyzing the formation of highly reactive hydroxyl radicals through the Fenton reaction, thereby leading to intensified oxidative damage to cardiomyocytes [62,117].

Doxorubicin-induced oxidative stress not only affects cellular integrity but also compromises mitochondrial function and promotes the release of pro-apoptotic factors, ultimately triggering programmed cell death pathways [118,119,120], directly contributing to cardiac dysfunction observed in doxorubicin treatment [121,122].

In addition to cell death via apoptosis, doxorubicin-induced oxidative stress has implications for endothelial function, promoting inflammation and further damaging cardiac tissue. Doxorubicin can induce a cascade of inflammatory responses, activating transcription factors such as NF-κB and leading to increased synthesis of various pro-inflammatory cytokines, including TNF-α [123,124,125]. This inflammatory environment exacerbates cardiac injury and contributes to the progressive nature of doxorubicin-induced cardiomyopathy [24,126,127,128,129,130].

To combat oxidative stress, various antioxidant-based therapies have been explored. Agents targeting the enhancement of cellular defense pathways against oxidative stress, such as the Nrf2-related antioxidant system, are being investigated [131,132]. However, despite the promise they present, clinical efficacy has often been limited, indicating a need for continued research into more effective cardioprotective strategies [133]. The complexity of doxorubicin-induced cardiotoxicity involves multifactorial mechanisms converging to form a detrimental cycle of oxidative stress, apoptosis, and inflammation, leading to long-term cardiac damage. Understanding these pathways can guide the development of targeted interventions aimed at mitigating cardiotoxicity while allowing for effective cancer treatment. Therefore, the exploration of novel pharmacological approaches and lifestyle modifications that may enhance the antioxidant capacity in cardiomyocytes is essential for improving patient outcomes.

## 7. Doxorubicin and Calcium in Cardiomyocytes

Another mechanism contributing to doxorubicin-induced cardiotoxicity involves the dysregulation of calcium ions (Ca^2+^) within cardiomyocytes. Doxorubicin has been shown to lead to intracellular calcium overload, primarily resulting from alterations in calcium-handling proteins and mechanisms that govern intracellular calcium levels [134]. This overload occurs when doxorubicin impairs critical calcium channels and pumps, such as the sarco-endoplasmic reticulum calcium ATPase (SERCA) and the sodium–calcium exchanger (NCX). The compromised function of these proteins disrupts the fine-tuned regulation of calcium influx and efflux, leading to a rise in the concentration of intracellular calcium [135]. Elevated intracellular calcium can subsequently impact mitochondrial calcium levels, leading to mitochondrial dysfunction and further cardiomyocyte injury [136].

The intricate relationship between calcium dysregulation and oxidative stress is particularly noteworthy, as it amplifies the cardiotoxic effects of doxorubicin. Increased intracellular calcium promotes the generation of more ROS and consequent mitochondrial stress, creating a feedback loop that exacerbates cellular injury [137,138]. For instance, elevated calcium levels contribute to the opening of MPTPs, which allow pro-apoptotic factors like cytochrome c to be released from the mitochondria, leading to apoptosis [139]. Moreover, calcium overload can disrupt the contractile function of cardiomyocytes, contributing to heart failure and other cardiac disorders commonly associated with doxorubicin treatment [133,140].

Furthermore, doxorubicin-induced dysregulation of calcium handling is not confined to acute effects. Chronic exposure to this chemotherapeutic agent has been shown to induce long-term alterations in calcium signaling pathways, increasing the risk of arrhythmias and other cardiac complications [140]. Alterations in calcium-handling proteins, such as ryanodine receptors and phospholamban, have been documented in studies where doxorubicin-treated cells exhibit impaired calcium release and relaxation dynamics [141,142]. This dysregulation can impact excitation–contraction coupling, leading to reduced contractility and detrimental effects on cardiac function.

Despite ongoing research into calcium-handling proteins as potential therapeutic targets, effective strategies to mitigate doxorubicin-induced calcium dysregulation remain limited [135]. The understanding of how to restore proper calcium homeostasis in the context of anthracycline therapy holds promise for improving outcomes in patients undergoing doxorubicin treatment [143]. Recent studies have explored and continue to investigate pharmacological agents that can enhance calcium handling and mitochondrial protection as adjuncts to doxorubicin therapy, aiming to ameliorate the cardiotoxic effects experienced by patient cohorts [136,138].

## 8. Mitochondrial Dysfunction in Doxorubicin-Treated Hearts

Mitochondrial injury is central to the cardiotoxic effects of doxorubicin. Doxorubicin disrupts mitochondrial DNA (mtDNA), impairing the electron transport chain and reducing ATP synthesis. The resulting alterations lead to significant energy deprivation, which is particularly detrimental in high-demand tissues like the heart [144]. In cardiomyocytes, energy depletion severely compromises contractile function and overall cellular integrity. Furthermore, the loss of mitochondrial membrane potential facilitates the release of cytochrome c into the cytosol, triggering the activation of caspases that mediate apoptosis [145]. Characteristic histological findings in doxorubicin-treated hearts include swollen mitochondria with disrupted cristae, which are indicative of cellular distress and injury [146].

**Figure 1:**
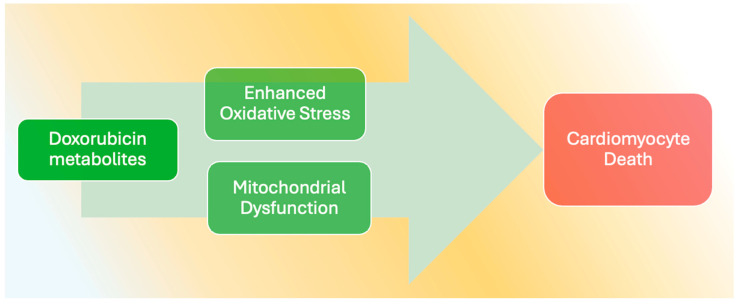
Accumulation of doxorubicin metabolites in the heart enhances oxidative stress via increased production of reactive oxygen species and mitochondrial dysfunction. These mechanisms can ultimately lead to cardiomyocyte injury, cardiac adverse remodeling, and, eventually, heart failure.

Doxorubicin also impairs mitophagy, the process by which damaged or dysfunctional mitochondria are selectively degraded. This impairment can lead to the accumulation of defective mitochondria, further exacerbating oxidative stress and cellular death [147]. The loss of effective mitophagy may be linked to alterations in the expression of mitochondrial biogenesis regulators like PGC-1α, which is essential for maintaining mitochondrial function and homeostasis [69]. Recent research highlights the intricate relationship between mitochondrial dysfunction and the propensity for oxidative damage in cardiomyocytes (Figure 1), reinforcing the hypothesis that targeting mitochondrial pathways may offer therapeutic benefits [148].

Moreover, additional mitochondrial alterations in response to doxorubicin include abnormal dynamics characterized by increased fission and reduced fusion, contributing to a fragmented mitochondrial network [69]. This altered architecture is detrimental to mitochondrial function as it affects the organelles’ ability to produce ATP efficiently and respond to metabolic demands during stress. The implications of such mitochondrial disruptions extend beyond immediate cellular pathways, potentially influencing broader cardiac health by affecting myocardial remodeling and function post-chemotherapy [149].

With the increasing awareness of the pivotal role of mitochondria in doxorubicin-induced cardiotoxicity, therapeutic strategies focusing on mitochondrial protection have emerged as a promising approach. Agents that enhance mitochondrial biogenesis or promote mitophagy might mitigate the cardiotoxic impacts of doxorubicin, thereby preserving cardiac function during cancer treatment [150]. These strategies, combined with ongoing research into the molecular pathways involved in mitochondrial injury, hold potential for improving the safety and efficacy of doxorubicin therapy in cancer patients.

## 9. Inflammatory Pathways and Immune Modulation

Inflammatory cascades are increasingly recognized as significant contributors to doxorubicin-induced cardiac damage. Doxorubicin activates innate immune signaling pathways, particularly through Toll-like receptors (TLRs), leading to enhanced production of pro-inflammatory cytokines such as tumor necrosis factor-alpha (TNF-α), interleukin-6 (IL-6), and interleukin-1 beta (IL-1β) [151,152]. This activation of TLRs plays a pivotal role in the inflammatory response, initiating cascades that result in further cardiomyocyte damage [153].

One particularly notable consequence of doxorubicin treatment is the activation of the NLRP3 inflammasome, which is integral to the inflammatory response [154]. This inflammasome triggers caspase-1 activation, leading to a form of cell death known as pyroptosis, characterized by cell swelling and lysis [155]. Pyroptosis releases additional inflammatory cytokines and damage-associated molecular patterns (DAMPs) into the extracellular environment, further propagating cardiomyocyte injury and promoting fibrosis [156,157].

Increased immune cell infiltration, particularly by macrophages, correlates with the progression to heart failure, suggesting that the inflammatory milieu contributes significantly to the deterioration of cardiac function [152,153,158,159,160]. Enhanced TLR signaling in macrophages is linked with the secretion of cytokines that exacerbate inflammation, leading to a cycle of tissue damage and repair that ultimately fails to restore normal cardiac function [161]. Notably, the TLR4 signaling pathway has emerged as a critical mediator of the inflammatory response in the context of doxorubicin-induced cardiotoxicity, mediating the production of both pro-inflammatory cytokines and ROS [151,152].

Targeting these inflammatory pathways may represent a novel therapeutic avenue for mitigating the cardiotoxic effects of doxorubicin. Interventions aimed at blocking TLR signaling or inhibiting downstream inflammatory mediators can significantly reduce cardiac damage associated with doxorubicin treatment [156]. For instance, the modulation of TLR4 signaling through pharmacological agents has demonstrated promising cardioprotective effects by reducing inflammatory cytokine levels and preserving cardiac function [156,162].

Overall, the recognition of inflammatory pathways as key players in doxorubicin-induced cardiotoxicity highlights the need for further exploration of therapies targeting the innate immune response. Addressing the inflammatory component of cardiotoxicity could not only improve patient outcomes but also enhance the overall efficacy of doxorubicin in cancer treatment.

## 10. Genetic and Epigenetic Susceptibility Factors

Genetic and epigenetic susceptibility factors play crucial roles in determining individual vulnerability to doxorubicin cardiotoxicity. Several genetic predictors have been identified, including polymorphisms in genes encoding carbonyl reductases, NAD(P)H oxidase subunits, and ATP-binding cassette (ABC) transporters, which impact the metabolism and efflux of doxorubicin, thereby influencing its efficacy and toxicity profiles. Notably, variants in the ABCB1 (MDR1) gene are particularly relevant, as increased expression of the ABCB1 protein correlates with diminished intracellular accumulation of doxorubicin. Though cardiac infiltration of doxorubicin decreases, this facilitates drug resistance in various cancers, including breast and liver cancers [163,164].

In addition to genetic factors, epigenetic modifications significantly affect gene expression in response to doxorubicin exposure. Doxorubicin induces specific changes in the transcription profiles of histone deacetylases (HDACs), which have been reported to be deregulated in treated cardiac tissues, thereby altering the chromatin landscape and gene expression related to cardiac function [67,165]. For example, the modification of histones, such as acetylation patterns, particularly at sites like H3K27, has been shown to act as a molecular switch that can activate cardiotoxicity-related genes [166]. Furthermore, global hypomethylation has been documented in doxorubicin-resistant cancer cell lines, suggesting that DNA methylation alterations contribute to therapeutic resistance [167].

Moreover, recent studies underscore the role of epigenetic remodeling mechanisms—including DNA methylation and histone modifications—in establishing a resistant phenotype to doxorubicin. For instance, histone-modifying enzymes such as DNA methyltransferases (DNMTs) and HDACs are implicated in the chemoresistance pathways by modulating the expression of anti-apoptotic genes that lead to resistance against drug-induced cell death when activated [168,169]. Such epigenetic alterations not only facilitate resistance but may also promote the development of cardiotoxicity by implicating genes involved in cardiac signaling pathways [170,171].

Understanding these genetic and epigenetic susceptibility factors is essential for developing personalized prevention strategies for doxorubicin-induced cardiotoxicity. This knowledge could inform the selection of patients predisposed to such adverse effects, allowing for tailored therapeutic approaches that mitigate risks while optimizing cancer treatment regimens [172].

## 11. Pediatric Versus Adult Cardiotoxicity Profiles

Children are particularly susceptible to the cardiotoxic effects of doxorubicin, resulting in significant long-term implications for cardiovascular health. Pediatric patients undergoing chemotherapy, especially those treated for conditions such as acute lymphoblastic leukemia, frequently exhibit delayed-onset cardiomyopathy that may not manifest until they reach adulthood. This delayed presentation complicates their health trajectory, as early detection and intervention for cardiotoxicity may be inadequate if monitoring does not continue beyond the immediate treatment phase [173,174,175].

The biological differences between pediatric and adult patients contribute to this heightened susceptibility [176]. Children undergo rapid cardiac development, which can exacerbate their response to doxorubicin. Additionally, although cardiac muscle cells in children possess a greater theoretical regenerative capacity, they are also more sensitive to oxidative stress and inflammatory responses induced by anthracycline exposure, leading to an increased risk of cardiomyopathy [174,177]. In contrast, adult patients often present with earlier symptoms of cardiotoxicity, primarily due to pre-existing comorbidities such as hypertension or metabolic syndrome, which exacerbate cardiac stress related to doxorubicin treatment [178,179].

To adequately understand and manage the cardiotoxic effects of doxorubicin in both pediatric and adult populations, longitudinal follow-up studies are essential. Research emphasizes the necessity for age-specific monitoring protocols and intervention strategies. For instance, periodic echocardiographic assessments can help identify subclinical cardiac dysfunction in children even before overt symptoms arise [180,181]. Furthermore, findings indicate that protective strategies, such as the administration of dexrazoxane, a chemoprotective drug, can mitigate the risk of cardiotoxicity, though their use must consider potential side effects [182,183]. Adaptive monitoring frameworks, tailored pharmacological interventions, and lifestyle modifications are vital for preventing cardiac morbidity in young cancer survivors as they age [181,184,185]. Thus, understanding these distinct profiles of cardiotoxicity across age groups is crucial for improving long-term cardiovascular health outcomes in both pediatric and adult cancer survivors.

## 12. Structural and Functional Cardiac Changes Detected by Imaging

Non-invasive imaging modalities have become indispensable in elucidating the cardiotoxic effects associated with doxorubicin treatment. Within this spectrum, echocardiography remains the foundational tool for assessing cardiac function, primarily by evaluating the left-ventricular ejection fraction (LVEF). However, advances in imaging technology have underscored the importance of global longitudinal strain (GLS), a measurement of the longitudinal shortening of the left ventricle, which offers enhanced sensitivity for detecting early myocardial dysfunction that may not be apparent through LVEF assessment alone [186,187]. While LVEF can remain within normal limits in the initial phases of treatment, an altered GLS can indicate subclinical cardiac impairment, thereby serving as a critical early warning sign of doxorubicin-induced cardiotoxicity [117,188].

Cardiac magnetic resonance imaging (MRI) has further expanded the diagnostic capabilities available to clinicians. It provides comprehensive structural insights, including the detection of myocardial edema, fibrotic changes detected through late gadolinium enhancement, and alterations in myocardial mass. This modality is particularly beneficial in assessing structural abnormalities associated with doxorubicin cardiomyopathy, as it provides unrivaled clarity of cardiac tissue and can visualize the extent of damage caused by this chemotherapeutic agent [187]. The superior ability of MRI to delineate soft tissue contrasts enhances its specificity in diagnosing cardiac conditions, thereby enabling timely interventions.

Additionally, nuclear imaging techniques such as multigated acquisition (MUGA) scans have historically played a role in monitoring cardiac function in patients receiving anthracyclines. While MUGA scans are useful, they are gradually being eclipsed by more sophisticated imaging modalities due to their limitations in spatial resolution and the comprehensive information provided by MRI and advanced echocardiographic techniques [187,189]. Given the implications of doxorubicin-induced cardiotoxicity, the integration of these imaging modalities is crucial for the early identification of patients at risk, allowing for timely therapeutic adjustments and potentially mitigating long-term cardiovascular complications [188,190].

In sum, the utilization of multimodal imaging approaches—including echocardiography, cardiac MRI, and nuclear imaging—facilitates a robust assessment of the structural and functional cardiac changes induced by doxorubicin. This comprehensive imaging strategy enhances our understanding of the cardiotoxic potential of doxorubicin and informs clinical decision-making to optimize patient outcomes [187,191].

## 13. Biomarkers of Cardiotoxicity in Doxorubicin-Treated Patients

Biomarkers of cardiotoxicity present a valuable, non-invasive approach for early detection of myocardial injury in patients undergoing doxorubicin therapy. Among the most established cardiac biomarkers, troponins—particularly high-sensitivity assays—have been shown to be predictive of long-term LVEF decline when elevated during treatment [192,193]. These biomarkers facilitate the identification of patients at increased risk for cardiac complications, enabling clinical teams to initiate preventive strategies or modify treatment regimens accordingly [192].

N-terminal pro-B-type natriuretic peptide (NT-proBNP) levels serve as another significant indicator, reflecting ventricular wall stress and correlating with symptomatic heart failure [194]. Elevated NT-proBNP is frequently observed in patients experiencing doxorubicin-induced cardiac dysfunction, and its measurement can provide insights into the hemodynamic changes occurring as a result of anthracycline therapy [194]. Importantly, high NT-proBNP levels have been consistently linked with adverse outcomes, highlighting their relevance in monitoring patients at risk for chemotherapy-related cardiac dysfunction [195].

Recent investigations continue to explore an array of novel biomarkers beyond traditional measures as well, such as galectin-3 and circulating microRNAs. For instance, microRNA-1 and microRNA-133b have emerged as potential biomarkers for early detection of myocyte injury and in the assessment of recovery from cardiotoxicity [196]. These circulating microRNAs reflect the complex interplay between myocardial stress and inflammation contributing to heart failure mechanisms [196]. Serial measurements of these biomarkers may not only enhance risk stratification but also guide treatment decisions, potentially leading to improved long-term outcomes in patients undergoing anthracycline therapy [192,197]. Most recently, specific microRNA combinations were shown to confer protection to cardiomyocytes against doxorubicin-induced toxicity in vitro; the most potent pair (miR-222 and miR-455) appeared to exert a synergistic effect [198].

The integration of these biomarkers into clinical practice can facilitate a more comprehensive assessment of cardiotoxicity during doxorubicin treatment, ultimately allowing healthcare providers to more effectively monitor patients and personalize therapeutic approaches [192,195]. As the field advances, continued research focusing on the validation and clinical application of these emerging biomarkers is essential for enhancing patient safety and optimizing cancer treatment strategies.

## 14. Endothelial Dysfunction

Vascular dysfunction and endothelial injury are significant complications associated with doxorubicin therapy, contributing to the overall cardiotoxicity experienced by patients undergoing treatment. Doxorubicin induces direct myocyte toxicity and thus compromises vascular integrity and endothelial function, leading to various cardiovascular repercussions.

When endothelial cells are exposed to doxorubicin, they exhibit increased permeability characterized by a disruption in tight junctions, resulting in enhanced trans-endothelial migration of fluids and leukocytes. This alteration is associated with the upregulation of adhesion molecules, such as VCAM-1 and ICAM-1, facilitating leukocyte adhesion to the endothelial surface [199]. Consequently, this promotes microvascular dysfunction and can further precipitate ischemia and tissue hypoxia, particularly in the myocardium where adequate perfusion is crucial for maintaining cardiac function [200].

In addition to increased permeability, doxorubicin significantly impairs nitric oxide (NO) production, primarily by affecting endothelial nitric oxide synthase (eNOS) [201]. Under normal conditions, eNOS generates NO, which exerts vasodilatory effects and maintains vascular homeostasis. However, doxorubicin reduces NO availability and shifts eNOS activity toward the production of ROS, thereby exacerbating oxidative stress and further compromising endothelial function [202,203]. This imbalance between NO and ROS is fundamental in the pathogenesis of endothelial dysfunction observed in cancer patients treated with anthracyclines.

Capillary rarefaction has also been documented in histopathological studies involving doxorubicin-treated hearts [204]. This reduction in capillary density contributes to impaired myocardial perfusion and inadequate supply of oxygen and nutrients to cardiac tissues, which are already stressed due to the cytotoxic effects of the drug. The persistence of vascular dysfunction can hinder the healing process following injury and may lead to chronic conditions such as heart failure and progressive cardiomyopathy [205].

Mounting evidence indicates that oxidative stress induced by doxorubicin leads to mitochondrial dysfunction and ultimately apoptosis within the endothelium [200,206]. Endothelial cell death diminishes the protective functions of the endothelial layer, including its role in regulating vascular tone and barrier integrity. Moreover, endothelial cell apoptosis can trigger inflammatory pathways, leading to a cascade of local and systemic effects that perpetuate the cycle of cardiac damage and exacerbate cardiovascular disease [202,207,208].

Recent studies have explored the potential therapeutic roles of various compounds in mitigating doxorubicin-induced endothelial injury. For instance, vitamin D has been shown to protect against endothelial dysfunction by attenuating oxidative stress and promoting the survival of endothelial cells exposed to doxorubicin [209]. Additionally, novel approaches such as the use of mesenchymal stem cells to support endothelial regeneration and improve microvascular recovery post-chemotherapy are currently under investigation [210].

Hence, cardiotoxicity associated with doxorubicin therapy is significantly influenced by vascular dysfunction and endothelial injury. The impact of doxorubicin on endothelial cells includes increased permeability, diminished NO production, and enhanced oxidative stress, all of which contribute to compromised vascular function and myocardial perfusion. As the understanding of these mechanisms evolves, targeted interventions aimed at preserving endothelial integrity and function hold promise for reducing the cardiovascular burdens of doxorubicin therapy in cancer patients.

## 15. Preclinical Models

Several animal models are extensively utilized to study the mechanisms of doxorubicin-induced cardiotoxicity, as well as potential therapeutic interventions.

The most frequently used animal models include rodent species, primarily rats and mice, due to their manageable size, ease of handling, and genetic manipulation capabilities. Among these, the rat model is particularly advantageous for studying doxorubicin-induced cardiomyopathy because of the physiological and anatomical similarities with humans. Administering doxorubicin via intraperitoneal injection results in significant cardiac dysfunction, which can be quantified by echocardiographic measures such as the left-ventricular ejection fraction and fractional shortening [211,212,213]. Histopathological examinations reveal hypertrophy, myocardial fibrosis, and apoptotic changes in cardiac myocytes, mirroring the clinical manifestations in humans [64,214,215].

Mouse models are also extensively used because they allow for genetic modifications, facilitating the study of underlying genetic susceptibility and the effect of various pharmacological interventions on cardiac injury due to doxorubicin [216,217]. For example, specific strains such as the SCID (severe combined immunodeficient) mouse have been utilized to investigate the protective effects of potential cardioprotective agents, including stem cell therapies and other pharmacological compounds [218,219]. Moreover, the administration of doxorubicin in mice has been shown to induce significant oxidative stress and alterations in intracellular calcium regulation, which can contribute to myocyte apoptosis and impaired contractility [220].

On the other hand, zebrafish models have emerged as novel alternatives due to their rapid development, transparency during embryonic stages, and amenability to high-throughput screening approaches. Doxorubicin-induced cardiomyopathy can be recapitulated in adult zebrafish, providing a unique platform for genetic screenings and drug testing [118,221]. The model showcases morphological changes similar to mammalian systems, allowing for the evaluation of cardiomyocyte function and histological assessment of cardiac tissue integrity [222].

Larger animals have also been used, albeit less frequently. Porcine models have shown promise, as they closely resemble human heart anatomy and physiology, making them suitable for studying the long-term effects of doxorubicin [217,223,224,225].

While rat and mouse models remain predominant in studying doxorubicin-induced cardiomyopathy due to their genetic and physiological advantages, zebrafish and larger animal models provide complementary insights into heart failure mechanisms and potential therapeutic strategies. Continued refinement of these models will enhance the understanding of doxorubicin cardiotoxicity and underpin the development of effective cardioprotective interventions.

## 16. Cardioprotective Strategies and Interventions

Much effort has been made to prevent doxorubicin cardiotoxicity, but according to the American Society of Clinical Oncology (*ASCO*), dexrazoxane remains the only current option to attempt to counteract doxorubicin cardiotoxicity [226], acting via its iron-chelating ability; however, the clinical use of dexrazoxane is limited by its interference with the chemotherapeutic activity of doxorubicin and major concerns regarding the increased incidence of secondary malignancies observed in cancer survivors following dexrazoxane treatment [5,227,228]. A report of the European Medicines Agency (*EMA*) has explicitly recommended several restrictions on dexrazoxane use in both children and adults with cancer [183]. Other treatments have not succeeded in eliminating the cardiac toxicity of doxorubicin [3,4,5,229,230]. Current oncology guidelines recommend limiting the total cumulative dose of doxorubicin to 450–550 mg/m^2^; however, this approach limits the effectiveness of anticancer treatment [231]. Therefore, new strategies to counteract the development of heart failure caused by doxorubicin are urgently needed. Liposomal formulations of doxorubicin could reduce myocardial exposure by altering tissue distribution. Pharmacologic agents such as beta-blockers, ACE inhibitors, and statins have demonstrated protective effects in clinical and preclinical studies. Antioxidants and mitochondrial-targeted therapies are also under active investigation. However, balancing cardioprotection with oncologic efficacy remains a critical challenge.

## 17. Monitoring Guidelines and Risk Stratification

Monitoring guidelines for patients undergoing anthracycline therapy, particularly with doxorubicin, have been developed by key professional societies, notably the ASCO and the European Society of Cardiology (*ESC*). These guidelines are essential for identifying patients at risk for cardiotoxicity and for implementing appropriate surveillance strategies throughout the course of treatment.

The *ASCO* and *ESC* guidelines emphasize the usefulness of a baseline assessment of cardiac function prior to the initiation of anthracycline therapy. This assessment typically includes an echocardiogram or other imaging studies to establish a reference LVEF [232,233]. Early identification of pre-existing cardiac conditions allows for informed decisions about treatment regimens, including potential modifications to dosages or scheduling.

According to the guidelines, regular monitoring of cardiac function during and after treatment is recommended, particularly for high-risk individuals. Surveillance often involves periodic echocardiograms, which enable the identification of changes in LVEF that may indicate the onset of cardiotoxicity. The guidelines also suggest incorporating advanced imaging techniques, such as speckle tracking echocardiography, to evaluate GLS and detect subclinical left-ventricular dysfunction earlier than LVEF alone [234,235].

Risk stratification is a crucial component of the monitoring strategy outlined in both the *ASCO* and *ESC* guidelines. Various factors are taken into account, including cumulative doxorubicin dose, patient age, presence of prior cardiovascular disease, and specific genetic markers that could predispose patients to increased risk of cardiotoxicity [232,236]. This stratification enables clinicians to tailor monitoring and therapeutic strategies, focusing on individuals at the highest risk of developing cardiac complications.

The guidelines advocate for the integration of cardiac biomarkers, such as troponins and NT-proBNP, into routine monitoring. Elevated troponin levels are indicative of cardiac myocyte injury, while NT-proBNP reflects ventricular wall stress and can correlate with heart failure [232]. These biomarkers, measured serially, provide additional insight into the patient’s cardiac status and facilitate timely interventions. The combined approach of imaging and biomarker assessment offers a more comprehensive understanding of the patient’s cardiac health. The concept of personalized monitoring protocols is a fundamental theme in the *ASCO* and *ESC* guidelines. By considering individual patient risks, including genetic predispositions and comorbidities, healthcare providers can develop tailored monitoring plans that optimize both cardiac safety and cancer treatment efficacy [237]. Such individualized strategies are vital for managing doxorubicin therapy, as they aim not only to mitigate cardiotoxicity but also to preserve the intended antitumor efficacy. The collaborative guidelines provided by *ASCO* and *ESC* for monitoring cardiac function during anthracycline therapy offer a robust framework for risk assessment and management. Through baseline evaluations, periodic surveillance, risk stratification, and biomarker integration, these guidelines aim to enhance early detection of cardiotoxicity and improve patient outcomes. Ongoing research and clinical experience will continue to refine these monitoring strategies, making them more effective in addressing the challenges associated with doxorubicin-induced cardiotoxicity.

## 18. Conclusions and Future Directions

Doxorubicin remains a cornerstone of cancer therapy due to its efficacy against various malignancies; however, its cardiotoxic potential necessitates vigilant monitoring and intervention to mitigate adverse cardiovascular effects. Significant advances have been made in the fields of imaging, biomarkers, and genomics, enhancing our understanding of the underlying mechanisms and risk factors associated with doxorubicin-induced cardiotoxicity. However, notable gaps persist in our ability to prevent and reverse cardiac injury effectively [238,239]. Recent advancements in cardiac imaging, such as echocardiography and cardiac MRI, have been pivotal in providing insights into cardiac structure and function during doxorubicin therapy. These modalities facilitate the early detection of cardiac dysfunction, which is critical because established morphological changes often occur after significant cardiac damage has already transpired [240]. Additionally, biomarkers like high-sensitivity troponins and NT-proBNP are proving to be valuable tools for ongoing monitoring of cardiac integrity, as they can indicate myocardial injury long before changes in ejection fraction take place [241]. Genomic and proteomic studies aimed at advancing our comprehension of individual susceptibilities to cardiovascular complications have revealed specific genetic markers associated with increased risk for doxorubicin-induced cardiotoxicity [242,243]. However, the practical integration of these genetic insights into routine clinical practice remains a challenge, as risk stratification models must encompass not only genetic factors but also cumulative dose, age, and pre-existing cardiovascular disease [244]. Despite these advances, our ability to prevent and effectively treat cardiac injury related to doxorubicin therapy remains limited. Current treatment strategies often focus on managing symptoms rather than proactively preventing cardiac dysfunction.

**Figure 2:**
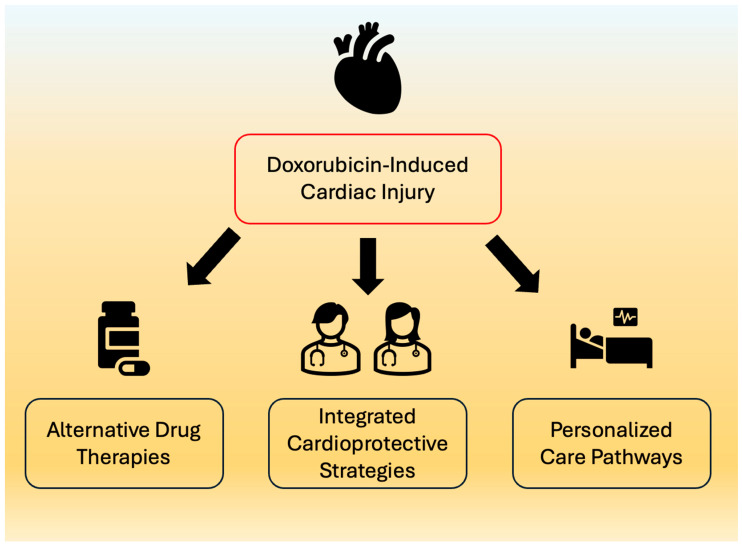
Future management of doxorubicin-induced cardiac injury. Forthcoming research and clinical strategies should prioritize the development of safe alternative drug therapies, integrated cardioprotective care models such as cardio-oncology, and personalized prevention and treatment pathways for patients.

To fill these gaps, future research should prioritize the development of safer doxorubicin analogs that retain antitumor efficacy while minimizing cardiac risks. Additionally, refining and incorporating these novel cardioprotective strategies into treatment protocols will be crucial (Figure 2); for instance, targeted therapies that reduce oxidative damage or promote endothelial healing could substantially mitigate the vascular injuries associated with doxorubicin [245,246,247,248,249,250].

Implementing personalized approaches to survivorship care is paramount to addressing the unique risk profiles of patients undergoing chemotherapy. Integrated care models, such as cardio-oncology, which combine oncology and cardiology expertise, will play an essential role in optimizing outcomes for cancer patients, enabling the identification of those at heightened risk for developing cardiac complications while simultaneously managing their oncological treatments.

In summary, while doxorubicin remains a foundational component of cancer therapy, vigilant monitoring and targeted interventions are essential to mitigate its cardiotoxic risks. Advances in imaging, biomarkers, and genomics have enhanced our understanding of the mechanisms underpinning doxorubicin-induced cardiac injury. Nevertheless, the healthcare community must confront existing challenges in prevention and treatment through further research focusing on safer drug alternatives, innovative cardioprotective strategies, and personalized care pathways. The integration of cardiac expertise into oncology care is vital for optimizing the health and quality of life of patients with cancer.

## Data Availability

Not applicable.
