# Correction: Bhutani et al. Doxorubicin-Induced Cardiotoxicity: A Comprehensive Update. *J. Cardiovasc. Dev. Dis.* 2025, *12*, 207

**DOI:** 10.3390/jcdd12070242

**Published:** 2025-06-25

**Authors:** Vasvi Bhutani, Fahimeh Varzideh, Scott Wilson, Urna Kansakar, Stanislovas S. Jankauskas, Gaetano Santulli

**Affiliations:** 1 Department of Medicine (Division of Cardiology), Wilf Family Cardiovascular Research Institute, Einstein Institute for Aging Research, Albert Einstein College of Medicine, New York City, NY 10461, USA; 2 International Translational Research and Medical Education (*ITME*) Consortium, Academic Research Unit, Department of Advanced Biomedical Sciences, “Federico II” University, 80131 Naples, Italy; 3 Department of Molecular Pharmacology, Einstein-Mount Sinai Diabetes Research Center (*ES-DRC*), Einstein Institute for Neuroimmunology and Inflammation (*INI*), Fleischer Institute for Diabetes and Metabolism (*FIDAM*), Albert Einstein College of Medicine, New York City, NY 10461, USA

In the original publication [1], references [118] and [221] are incorrect, the correction version are shown below.

Reference [118]: Lam, P.Y.; Kutchukian, P.; Anand, R.; Imbriglio, J.; Andrews, C.; Padilla, H.; Vohra, A.; Lane, S.; Parker, D.L., Jr.; Cornella Taracido, I.; et al. Cyp1 Inhibition Prevents Doxorubicin-Induced Cardiomyopathy in a Zebrafish Heart-Failure Model. *Chembiochem* **2020**, *21*, 1905–1910.

Reference [221]: Ma, X.; Ding, Y.; Wang, Y.; Xu, X. A Doxorubicin-Induced Cardiomyopathy Model in Adult Zebrafish. *J. Vis. Exp*. **2018**, *136*, 57567.

A new reference is added as [249].

Reference [249]: Nie, M.; Lei, D.; Liu, Z.; Zeng, K.; Liang, X.; Huang, P.; Wang, Y.; Sun, P.; Yang, H.; Liu, P.; et al. Cardiomyocyte-Localized CCDC25 Senses NET DNA to Promote Doxorubicin Cardiotoxicity by Activating Autophagic Flux. *Nat. Cancer*, 2025; *in press*.

The original reference [249] has been renumbered as [250]. The citation of new reference [249] has now been inserted in Section 18 and should read:

To fill these gaps, future research should prioritize the development of safer doxorubicin analogs that retain antitumor efficacy while minimizing cardiac risks. Additionally, refining and incorporating these novel cardioprotective strategies into treatment protocols will be crucial (Figure 2); for instance, targeted therapies that reduce oxidative damage or promote endothelial healing could substantially mitigate the vascular injuries associated with doxorubicin [245–250].

The author would like to apologize for any inconvenience caused to the readers by these changes. The authors state that the scientific conclusions are unaffected. This correction was approved by the Academic Editor. The original publication has also been updated.
